# Pupil size response within direct and random exploration and exploitation behaviors selectively reflects value of control

**DOI:** 10.3389/fpsyg.2026.1752586

**Published:** 2026-03-03

**Authors:** Gili Barkay, Shai Gabay, Uri Hertz

**Affiliations:** 1 School of Psychological Sciences, University of Haifa, Haifa, Israel; 2 The Institute of Information Processing and Decision Making, University of Haifa, Haifa, Israel; 3 Department of Cognitive Science, University of Haifa, Haifa, Israel

**Keywords:** exploration-exploitation, pupillometry, cognitive control, arousal, value of control

## Abstract

**Background:**

Balancing exploration and exploitation is central to adaptive decision-making and is thought to depend on interactions between arousal-related neuromodulation and strategic control. The present study examined how pupil-indexed arousal corresponds to different aspects of exploration and exploitation decisions.

**Method:**

We used the Horizon Task, which independently manipulated value of control through value uncertainty, information asymmetries, and choice horizon. Thirty-five participants completed 320 mini-games while pupil diameter was continuously recorded, with analyses focused on the first free-choice trial.

**Results:**

Behaviorally, participants exploited more when value gaps were larger, preferentially sampled the option with fewer prior observations and showed increased exploration in long-horizon conditions, where additional choices enabled the use of newly acquired information. These patterns replicate established patterns of directed and random exploration. Pupillary responses, however, showed a selective profile. For exploitative choices, though not for exploratory choices, pupil size increased when horizons were short and when value differences were small, indicating greater arousal during decisions with higher immediate importance or increased discrimination demands, reflecting increased value of control. Trial-by-trial analyses revealed sustained pre-decision modulation rather than discrete phasic peaks.

**Conclusion:**

Together, these findings allow integration of value of control approach and exploitative and exploratory control modes, indicating highlighting how strategic demands within each mode shape pupil-linked arousal.

## Introduction

Decision-makers face a fundamental challenge: should they exploit familiar, rewarding options or explore uncertain alternatives that might yield better long-term outcomes? (Cohen et al., 2007; Cogliati Dezza et al., 2017). This exploration - exploitation dilemma arises across species and contexts, from animal foraging to human strategic planning (Monk et al., 2018; Berger-Tal et al., 2014; Rich and Gureckis, 2018), yet questions persist regarding the psychological and neurobiological mechanisms underlying these competing drives (Berger-Tal et al., 2014; Mehlhorn et al., 2015).

Adaptive gain theory (AGT) provides a neurobiological framework for understanding the explore–exploit balance by linking behavioral modes to activity patterns in the locus coeruleus–norepinephrine (LC- NE) system (Aston-Jones and Cohen, 2005). According to AGT, the LC-NE system operates in two functionally distinct modes. In the phasic mode, the LC exhibits transient bursts of activity in response to task-relevant events, supporting focused attention and goal-directed behavior, conditions optimal for exploiting known reward sources. In the tonic mode, elevated baseline LC firing rates, reflecting a state of exploratory control - heightened arousal and promotion of exploration of alternative options, while lower tonic rates are associated with exploitative control, which is focused on choosing the known best option. Critically, these modes are not merely different levels of activation but correspond to qualitatively different patterns of neural activity that bias behavior toward exploitation or exploration, respectively.

Pupil diameter provides a noninvasive window into these LC-NE dynamics. Changes in pupil size closely track LC activity: baseline (average) pupil diameter reflects tonic LC firing, while transient pupil dilations index phasic LC responses (Joshi et al., 2016; Murphy et al., 2014). This correspondence allows pupillometry to test AGT’s core prediction that exploration and exploitation categorically distinct neuromodulatory states. Specifically, AGT predicts that exploration should be accompanied by larger tonic pupil size, reflecting sustained arousal in the tonic LC mode, whereas exploitation should be characterized by smaller baseline pupils with transient phasic dilations time-locked to decision-relevant events (Jepma and Nieuwenhuis, 2011). Consistent with this view, recent work of Fan et al. (2023), has demonstrated that baseline pupil size tracks total uncertainty in the environment and is associated with increased random exploration, suggesting that tonic arousal reflects a global state of uncertainty rather than specific decision demands (Fan et al., 2023).

While AGT framework links neuromodulatory activity to behavioral flexibility, previous findings revealed that exploration is not a unitary phenomenon. Wilson et al. (2014) distinguished directed exploration- deliberate information-seeking guided by uncertainty, from random exploration, which reflects stochastic choice variability unrelated to specific information goals. This functional distinction challenges AGT’s arousal-based account: directed exploration requires effortful goal maintenance and value integration, whereas random exploration may arise from diffuse arousal or decision noise. Yet these two forms of exploration exhibit fundamentally different behavioral pattern: directed exploration is modulated by information structure and planning opportunities, whereas random exploration scales with overall uncertainty regardless of whether the environment affords effective information use (Gershman, 2019).

This dissociation indicates that exploration cannot be fully captured by arousal fluctuations alone but depends on strategic control processes selectively recruited according to decision context. The Expected Value of Control (EVC) framework provides a complementary account, proposing that cognitive control is allocated when anticipated benefits, such as reducing uncertainty or optimizing future outcomes- outweigh intrinsic effort costs (Shenhav et al., 2013). Critically, EVC predicts that control deployment should scale with the prospective utility of investing effort in each decision rather than with exploration per se. This perspective suggests that exploration and exploitation behaviors may recruit control levels depending on strategic context, specifically, whether the decision environment affords sufficient opportunity to capitalize on acquired information (Sadeghiyeh et al., 2020; Frömer et al., 2021). Empirical evidence supports this prediction: Dubois and Hauser (2022) demonstrated that the planning horizon, the number of choices remaining in a task, dictates exploration strategy. They found that participants strategically increased random exploration only when the horizon was long (e.g., six choices), affording an opportunity to capitalize on the information, but relied less on this strategy when the horizon was short. This cost–benefit logic has been formalized computationally by Musslick et al. (2015), who demonstrated that the cognitive mechanisms governing goal-directed response selection determines when to deploy effortful control versus rely on stochastic processes.

Integrating AGT and EVC thus suggests that arousal and control jointly regulate adaptive decision-making: tonic arousal sets background readiness for behavioral flexibility, while strategic control is selectively recruited when decision context renders effortful processing worthwhile. Recent neurophysiological evidence supports this integrated view. Kozunova et al. (2022) using a continuous probabilistic learning task, provided direct support for this distinction. They operationalized exploitation as choosing the known high-payoff (HP) option after learning and directed exploration as choosing the known low-payoff (LP) option. They found that directed exploration elicited significantly larger phasic pupil dilations and slower response times compared to exploitation. This effect was absent during “random exploration” (choices made before learning), suggesting the pupil response tracked more than just uncertainty. Instead, the authors compellingly attribute the dilation to the cognitive conflict arising from intentionally overriding a strong, learned bias to exploit. However, both exploration and exploitation decisions happened within a continuous learning task, where exploitation reflects low expected value of control, while directed exploration reflects higher value of control. Shorter choice horizon, where the current choice cannot be used to inform future decisions, may reflect different values of control to exploration and exploitation.

This finding isolates a key component of strategic processing, linking pupil dilation to conflict-driven control. A key open question, however, is whether this pupillary response is specific to this form of internal conflict, or if it reflects a broader component of exploration, such as *decision uncertainty*. This distinction is central to integrating the EVC and AGT frameworks, particularly as other findings suggest pupil-linked arousal can be dissociated from effective control deployment. Frömer et al. (2021), for example, demonstrated that while neural markers of control (ERPs) scaled with reward and efficacy, pupillary responses showed a contrasting pattern: they were largest under low efficacy conditions. In that context, uncertainty was high, but strategic control was inefficacious. This suggests that pupil responses can track uncertainty-driven arousal even when strategic control is not effectively deployed.

**Figure 1:**
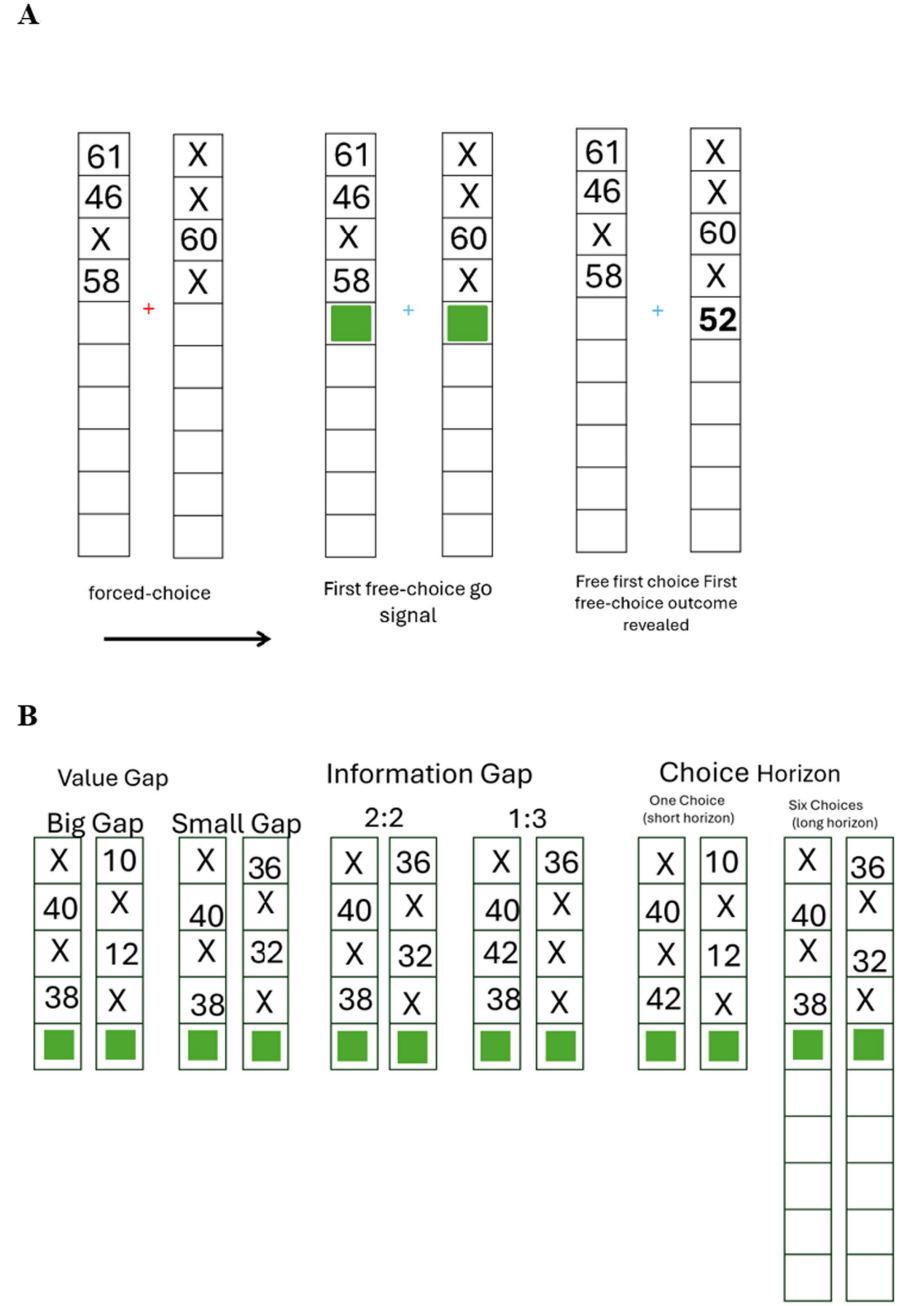
Experimental design: **(A)** Example trial sequence in the Horizon Task. The first four trials were forced-choice trials (left), where one choice was marked with X and the participants had to choose the other option and see its outcome. In free-choice trials a green square indicated that participants could freely choose between the two options. The selected option’s reward was then displayed (right). **(B)** Illustration of task paradigm and variables: The paradigm included 320 mini-games, that differed in three variables: value gap – the absolute difference in expected reward between options, information gap - imbalance in the number of samples from each option during the forced-choice session, and choice horizon - the number of remaining free-choice trials after the forced choice session. Green boxes indicate the first free choice, which was the focus of our analysis.

The present study examines how pupil-indexed arousal relates to exploration and exploitation under varying conditions of uncertainty, information structure, and planning opportunity. Using the Horizon Task, a multi-trial decision task in which participants choose between two options with different reward distributions (Figure 1). In this task, participants complete four forced-choice trials that provide initial information about each option’s payoff, followed by either one or six free-choice trials during which they can explore or exploit. We manipulate value differences, information asymmetries, and horizon length to differentially promote directed exploration, random exploration, and exploitation. AGT predicts that explorative control elicits elevated tonic arousal while exploitative control engages phasic responses. EVC predicts arousal scales with the strategic value of control deployment rather than choice type. In the horizon task, directed exploration is associated with the choice of low value option under long horizon conditions, while random exploration is associated with choosing the low value option under short horizon conditions. Exploitation is related to choosing the high-value option, but in this task the importance of exploitation changes. Specifically, when value differences are small (high uncertainty), exploitative choices require greater discriminative effort and carry higher impact on outcomes, increasing their expected value of control. Similarly, when horizons are short, exploitative decisions become more critical as there are no future opportunities to correct errors, further elevating their value of control. In contrast, exploration choices under long horizons may carry lower control demands because errors can be compensated for in subsequent trials, while exploratory choices under short horizons (random exploration) may reflect low-cost stochastic sampling rather than effortful strategic processing. Thus, integrating EVC consideration provides novel predictions for pupil diameter in exploration and exploitation decisions, predicting that pupil size should be larger (reflecting higher arousal and control investment) when the expected value of control is high, specifically, during exploitative choices under small value gaps or short horizons, and smaller when the expected value of control is low, as in exploratory choices where strategic demands are minimal. By measuring pupil dynamics across these conditions, this study tests whether arousal tracks exploratory versus exploitative choices or the contextual factors determining when effortful processing is warranted.

## Method

### Participants

Thirty-five participants between ages 18–41 (24 females, 13 males; *M* = 25.9, *SD* = 5.2) took part in the study. A sensitivity power analysis (*α* = 0.05, two-tailed, 80% power; Faul et al., 2009) indicated that with 35 participants the study was sufficiently powered to detect medium-sized effects (f ≈ 0.25, η^2^p ≈ 0.06) (Cohen, 2013) All had normal or corrected-to-normal vision and no history of neurological or psychiatric disorders. Participants were recruited through volunteer sampling within the academic institution and provided written informed consent in accordance with institutional guidelines. The experimental task lasted approximately 45 min, and participants received monetary compensation of $20 USD for their participation without any performance-based bonus. The study was approved by the institutional review board (IRB Approval code 252/22).

### Task and design

Participants completed an adapted version of the Horizon Task, designed to manipulate directed and random exploration. The task is programmed using MATLAB and Psychtoolbox 3.017. The experiment consisted of 320 games, divided into four blocks of 80 games each. Each game contained either 5 or 10 trials. On each trial, participants chose between two slot machine options, each delivering a reward between 1 and 100 points. Rewards were drawn from Gaussian distributions (SD = 8), with the mean value of one option set to either 40 or 60 and the other differing by 4, 8, 12, 20, or 30 points. These differences manipulated the expected value gap, such that smaller differences introduced greater uncertainty and encouraged exploration, while larger differences promoted exploitation.

The two choice options were visually identical and presented symmetrically to the left and right of a central fixation point. Choice and outcome history for each option remained visible within the corresponding display throughout the game. Each game began with four forced-choice trials, during which only one option was available at a time (Figure 1A). The unavailable option was marked with an “X” ensuring controlled exposure to outcome information while maintaining active task engagement. This phase-controlled participants’ initial information exposure prior to free choice. After each choice, the reward outcome was displayed on screen, along with a history of choices and rewards for both options within the current game. We manipulated three task variables, which captured distinct exploration- exploitation related dynamics (Figure 1B):

(1) Value gap: the absolute difference in expected reward between the two options. Larger gaps are associated with low uncertainty, whereas smaller gaps increase uncertainty.
(2) Information gap: the sampling imbalance during forced trials, defined by the number of prior samples from the higher-value option (+2 = more samples from the better option, −2 = more from the worse option, 0 = equal). Lower exposure to the superior option was expected to promote information-seeking via directed exploration.
(3) Choice horizon: the number of remaining free-choice trials (1 vs. 6). A longer horizon was expected to promote direct exploration by increasing the prospective utility of information, while a shorter horizon was expected to increase the importance of exploitation due to limited opportunity to benefit from newly acquired information. This variable was dummy-coded with the *long-horizon* condition (6 trials) serving as the reference category (coded as 0), and the *short-horizon* condition (1 trial) coded as 1.

### Procedure

Participants were first provided with on-screen illustrated instructions explaining the task structure, the fixed reward distributions within each game, and the goal of maximizing total points. Similar to Wilson et al. (2014), the instructions clarified that reward distributions remained constant within each game but varied across games, and that one option was always superior on average. Participants were informed that the initial trials in each game were forced-choice trials designed to provide information about the options before free choice. No separate practice block was administered prior to the experimental task; participants proceeded directly to the task following the instructions. Each game began with a fixation cross presented at the center of the screen. During the forced-choice phase, successive forced-choice trials were separated by brief inter-step intervals of 100 ms. Following the final forced-choice trial, the fixation display remained on screen for an additional 200 ms before its color changed, signaling the transition to the free-choice phase. The onset of the first free-choice trial occurred after a further 800 ms interval.

Following fixation, the choice display appeared and remained on screen until a response was made (self-paced- with a maximum allowed response time of 5,000 ms). During the free-choice phase, successive free-choice trials were separated by 500 ms intervals. After a choice, the selected option was highlighted and the numerical reward outcome was presented for a fixed interval, after which the next trial commenced. Between games, a blank screen was presented for 2000 ms before the onset of the next game. Throughout the task, pupil size was recorded continuously. To isolate anticipatory cognitive effort associated with exploratory behavior, pupillometry analyses focused exclusively on the first free-choice trial of each game. Pupil measures were extracted from the interval spanning choice onset until the motor response, allowing assessment of sustained decision-related arousal rather than stimulus-locked responses.

### Pupillometry recording and analysis

All behavioral and pupillometry data were processed and analyzed following a standardized pipeline. Pupil size data were recorded via the EyeLink 1,000 system at a sampling rate of 1,000 Hz. Preprocessing included blink detection, linear interpolation, and baseline correction. Trials with missing data were excluded. To ensure measurement accuracy and control for gaze position, the task was programmed to restrict responses unless the participant’s gaze was fixed at the center of the screen at the time of choice.

Pupil data were processed and visualized using Data Viewer software (version 4.1.211 Research Ltd., Ontario, Canada), which enabled blink detection verification, baseline correction, segmentation of trials, and extraction of tonic and phasic pupil measures. The primary tonic measure was defined as the average pupil size across the entire decision interval (from choice onset until the motor response) on the first free-choice trial of each game. Phasic analyses involved segmenting each trial into 100-ms bins spanning the 600 ms prior to the motor response (from Bin −6 to Bin 0) to examine the temporal dynamics of pupil-linked arousal, to capture the decision-related phasic burst, which is known to be time-locked to the motor response^36^.

The experimental task was programmed in MATLAB (version 2023a, MathWorks, Natick, MA, USA) using Psychtoolbox-3. All statistical analyses, including mixed-effects regression models for behavioral and pupillometry data, were conducted in SPSS (version 28, IBM Corp., Armonk, NY, USA).

All models included random intercepts for participants. More complex random-effects structures including random slopes for the experimental predictors were evaluated but did not converge reliably. Therefore, the reported results are based on the most parsimonious models that yielded stable estimates. Logistic mixed-effects models were used for the behavioral data to predict choice behavior on the first free-choice trial. For the primary pupillometry analysis, general linear mixed-effects models were applied. Critically, to test our main hypothesis, these models were conducted separately for exploitation trials (defined as choosing the higher-value option) and exploration trials (defined as choosing the lower-value option). In this analysis dependent variable is the pupil size during the period immediately preceding the motor response in the first free-choice trial of each game, reflecting anticipatory arousal. Predictor coding is consistent with the behavioral 1, except for Information gap, which reflects the symmetry of information between options (equal = 0; unequal = 1). These analyses were carried out in SPSS version 28.

## Results

All models included random intercepts for participants. More complex random-effects structures including random slopes for the experimental predictors were evaluated but did not converge reliably. Therefore, the reported results are based on the most parsimonious models that yielded stable estimates.

### Behavioral model

To examine the factors influencing participants’ choices on the first free-choice trial of each game, we conducted a logistic mixed-effects regression model. The model predicted the probability of choosing the higher-value option as a function of three fixed-effect predictors Value gap, Choice Horizon and Information gap (Figure 1B, see full description in methods).

The model revealed significant effects for all predictors. Participants were more likely to choose the higher-value option when the reward difference between options was larger (Value Gap: *β* = 0.019, *p* < 0.001), indicating increased exploitation under conditions of low uncertainty. A negative effect of Information Gap (*β* = −0.058, *p* = 0.009) revealed that participants tended to choose the higher-value option more often when they had received more prior information about it, whereas they were more likely to explore the lower-value option when it had been sampled more frequently that demonstrating a directed exploration strategy aimed at reducing uncertainty. Choice horizon also showed a significant positive effect (*β* = 0.167, *p* = 0.007), indicating that participants exploited more in short-horizon conditions (1 free trial), where the immediate payoff was crucial. Since the short horizon was dummy-coded as 1, the positive coefficient reflects a greater tendency to choose the higher-value option in that condition, compared to the long-horizon condition (6 trials), where exploration could support future gains (Table 1).

**Table 1:** Mixed-effects logistic regression results for first free-choice decisions.

Predictor	Coefficient *β*	Std. error	Exp (OR) (Coefficient)	CI for OR 95%	*p* value
Value gap (large)	0.019	0.0034	1.019	[1.012, 1.026]	**<0.001**
Information gap (positive)	−0.058	0.0223	0.943	[0.903, 0.985]	**0.009**
Choice horizon (short)	0.167	0.0623	1.182	[1.046, 1.335]	**0.007**

Logistic mixed-effects models predicting choice behavior during the first free-choice trial, conducted across all games. The model predicted the probability of choosing the higher-value option as a function of value gap, information gap, and choice horizon. **p < 0.01, ***p < 0.001. Models included random intercepts for participants.

### Average (tonic) pupil size

For the tonic analysis, pupil size was averaged across the entire response time of the first free-choice trial in each game, from the onset of choice presentation until the motor response. This measure captures overall anticipatory arousal during the decision process.

First, we examined whether pupil size was different between exploratory and explicatory decisions across all trials, using mixed-effects linear regression. We found that exploration decisions were associated with larger pupil size, but in marginal significance [*F* (1.5200) = 2.95, *β* = 19.62 *p* = 0.086]. This finding indicates that exploratory decisions were associated with higher arousal to some extent, in line with the AGT prediction of exploratory and exploitative control.

**Figure 2:**
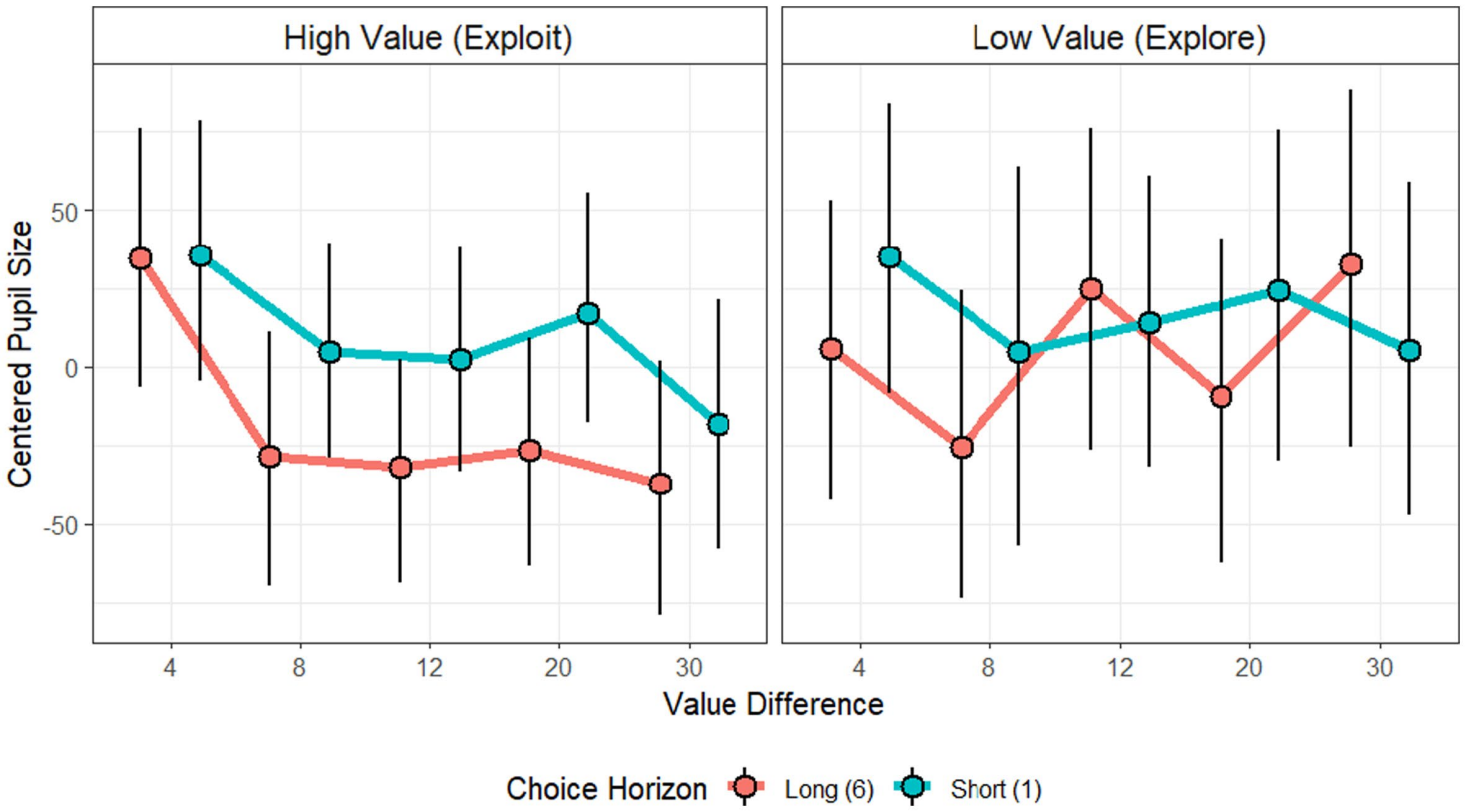
Pupil size during the experimental conditions. To demonstrate the effects observed in our statistical model, we plotted the pupil size (after removal of individual baseline) during exploit (left) and explore (right) choices, for short horizon (blue) and long horizon (red) conditions, in different value-gap choices. Exploratory choices were marked with somewhat larger pupil size, though not significantly so. In exploit decisions, pupil size decreased when uncertainty decreased (high value difference between the options) (*p* < 0.05) and was overall higher in short horizon choices than in long horizon choices (*p* < 0.05). Points indicate mean values and error bars indicate 95% bootstrap of the mean.

We then proceeded to analyze the effects of value gap, information gap and choice horizon on pupil size. To differentiate between their effects on exploration and exploitation, we carried out two independent analyses, one including only exploratory choices, and the other including exploitative choices (Figure 2). Table 2 presents the results of these models. For exploratory choices, none of the predictors significantly modulated pupil size (all *p* > 0.36), indicating that average pupil-indexed arousal did not vary with factors hypothesized to promote directed and random exploration. In contrast, for exploitative choices both Choice Horizon (*β* = −5.63, *p* = 0.025) and Value Gap (*β* = −1.58, *p* = 0.021) significantly predicted pupil size. Specifically, pupil size was larger in short-horizon trials (where only one decision remained) compared to long-horizon trials, indicating that the importance of the choice played an important role, in line with EVC account. In addition, pupil size increased when the value difference between options was small, indicating greater uncertainty, compared to when the difference was large and uncertainty diminished. This indicates that exploitation under low uncertainty is related to low arousal, but exploitation under high uncertainty is related to high arousal and task engagement, similarly, to predicted directed exploration in AGT. Finally, Information Gap did not significantly modulate pupil size in either exploration or exploitation contexts.

**Table 2:** Linear mixed-effects model results for pupil size by choice type.

Predictor	Coefficient β	Std. Error	CI 95%	*p* value
Exploration (lower-value option)				
Horizon	−3.07	3.38	[−9.69, 3.56]	0.364
Value gap	0.56	0.93	[−1.25, 2.38]	0.542
Info gap	−4.40	16.90	[−37.56, 28.75]	0.794
Exploitation (higher-value option)				
Horizon	−5.63	2.51	[−10.56, −0.71]	0.025*
Value gap	−1.58	0.68	[−2.92, −0.24]	0.021*
Info gap	8.31	12.56	[−16.33, 32.94]	0.509

Linear mixed-effects models predicting mean pupil size during the decision period, conducted separately for exploration (choosing the lower-value option) and exploitation (choosing the higher-value option). Significant effects (*p* < 0.05) are shown. Models included random intercepts for participants.

### Trial-by-trial binned pupil size

To identify the precise timing of pupillary effects, we conducted a binned - analysis of the pupil responses (i.e., phasic response) during the time leading to the decision point. To identify the precise timing of pupillary effects, we conducted a binned - analysis of the pupil responses (i.e., phasic response) during the time leading to the decision point. The group-level mean response time was 415.19 ms (SD = 122.66 ms). The majority of responses (87%) occurred within 700 ms prior to the choice, supporting the use of a 700-ms pre-response window for the time-bin analysis. Because many responses were faster, the number of observations contributing to each time bin was not uniform and decreased for bins farther from the response onset (see Supplementary materials for the RT distribution). Next, the 700 MS period preceding each motor response was segmented into seven successive 100 ms bins, aligned to the response onset (from Bin −6 to Bin 0).

Next, the 700 MS period preceding each motor response was segmented into seven successive 100 ms bins, aligned to the response onset (from Bin −6 to Bin 0). A separate linear mixed-effects model, similar to the one conducted for the average pupil size in the previous section, was fitted to all participants’ pupil msta within each time bin, with the same predictor structure as the tonic analysis (value gap, information gap, and choice horizon). This approach yielded a time course of beta (*β*) coefficients for each predictor, allowing us to identify when during the decision period each factor influenced pupil result. The full statistical tables for each bin are provided in the Supplementary Materials.

In addition to the beta coefficients, we examined model-based predicted pupil values (estimated marginal means) across time bins to facilitate interpretation of the direction and magnitude of pupil changes.

**Figure 3:**
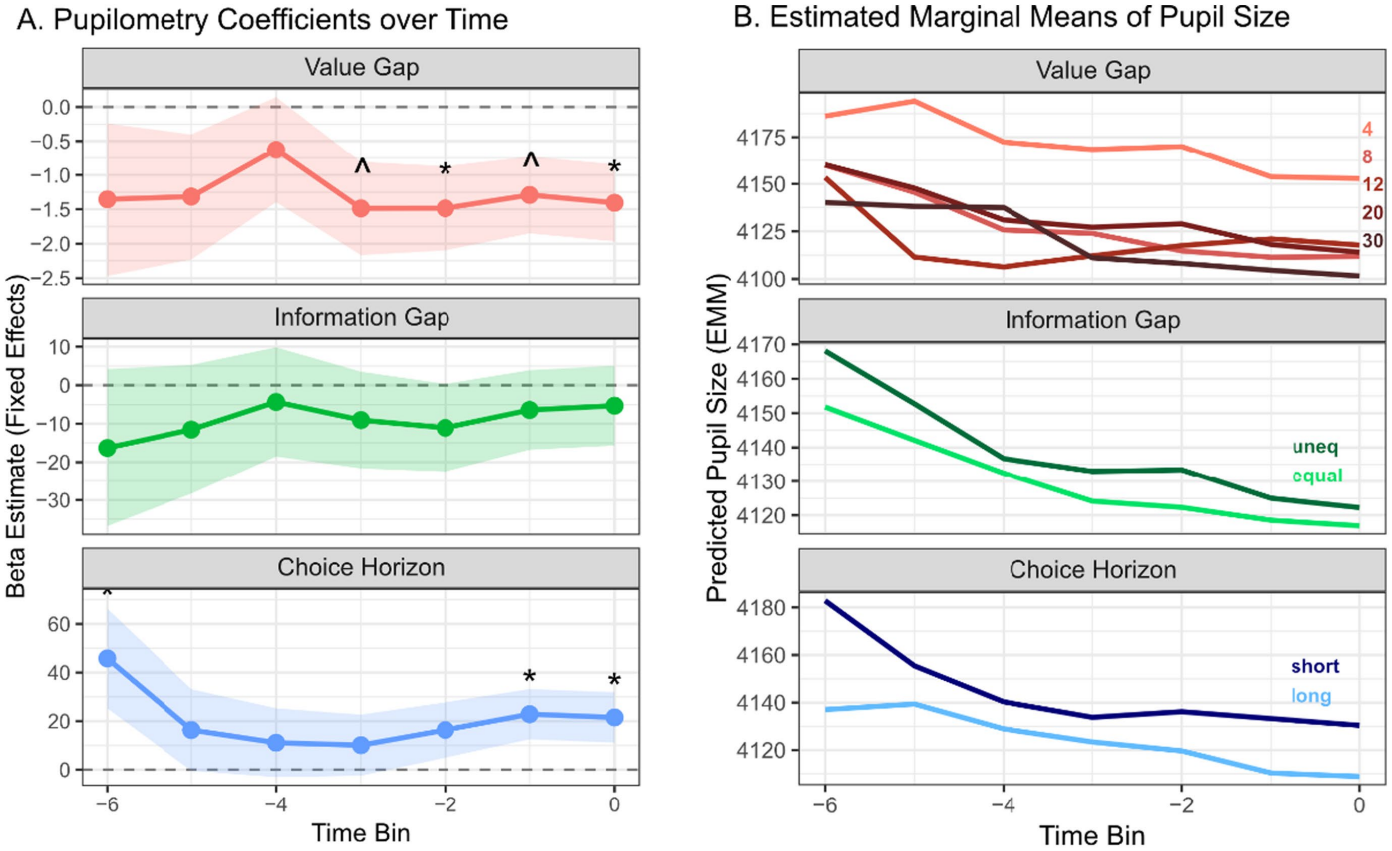
**(A)** Trial-by-trial analysis of pupil size across pre-decision time bins. The beta coefficients for predictors of pupil size in the 700 ms preceding the first free-choice decision, analyzed across seven consecutive 100 ms bins (Bin −6 to Bin 0) aligned to the motor response. Beta coefficients from linear mixed-effects models are shown in circles (connected with lines) for value gap (blue), choice horizon (red) and information gap (green). Shaded areas represent the standard error (SE), *p < 0.05, ^ marginal significance *p* < 0.08. **(B)** Model-based predicted pupil size (estimated marginal means) across the pre-decision time bins, separated for each condition (value-gap, information gap and choice horizon).

In Figure 3A, Value gap was negatively associated with pupil size across the decision window, with *β* coefficients consistently below zero. These negative values reached significance only in bin −2 (*β* = −1.20, *p* = 0.05) and 0 (*β* = −1.14, *p* = 0.048), showing reduced pupil size when the absolute difference between option values was larger. These results do not support a phasic effect of value-gap on pupil size, but a weak and consistent negative effect, similar to the marginal effect observed in the average pupil size analysis.

Choice horizon was a significant positive predictor of pupil size at bin −6 (*β* = 51.32, *p* = 0.016) and bin −1 (*β* = 21.80, *p* = 0.039), with a marginally significant effect at bin 0 (*β* = 18.25, *p* = 0.08). These results indicate larger pupil size in the short-horizon condition (1 free choice) compared to the long-horizon condition (6 free choices). However, the effects did not manifest as a discrete phasic peak, but rather as a more sustained modulation across the decision window.

Information gap was not significantly associated with pupil size at any point in the decision window (all *p* > 0.05). Coefficients were consistently negative across bins (ranging from *β* = −11.70 at bin −6 to *β* = 0.034 at bin 0), but none reached statistical significance. These findings indicate that sampling asymmetry (equal vs. unequal information) did not elicit reliable modulation of pupil-linked arousal, and no evidence for a phasic peak was observed.

The results from all time-bin analyses show that effects were somewhat more consistent immediately before the response, where they were similar in direction to the effects observed in the average pupil size analysis. This suggests that the observed pupil modulation reflects a sustained cognitive process leading up to the decision, rather than a discrete, transient response to an external stimulus. It also indicates that temporal dynamics along the time leading to decision may contribute to pupil size.

In Figure 3B, by using estimated marginal means, the predicted values clarify the direction and magnitude of pupil changes over time, demonstrating overall larger pupil size in the short-horizon condition relative to the long-horizon condition throughout the decision window. Alongside a gradual decrease in pupil size toward response execution. Importantly, this pattern confirms the absence of a discrete phasic peak and supports the interpretation of a sustained, context-dependent modulation of pupil size leading up to the decision.

## Discussion

The present investigation examined the relationship between pupillary responses and different types of control demands, associated with exploration and exploitation behavior. We used a decision-making task where the value of control was manipulated in different ways, including the level of uncertainty about the best option, the future consequences of the decision and the importance of current outcome. These allowed us to track subtle differences within exploration and exploitation decisions, and to examine how they bear common theories of task engagement. Behavioral analyses confirmed the importance of these factors in influencing participants’ choices. Participants adjusted their choices based on value uncertainty, consistently chose options with less prior information in asymmetrical-information conditions, and exhibited greater information-seeking behavior when more free choices remained (six vs. one). These patterns replicated Wilson et al. (2014) findings and confirmed the effectiveness of the experimental manipulations in eliciting exploration behavior.

Pupillary responses, however, revealed a more selective pattern. In line with AGT predictions, pupil size was somewhat larger in exploratory choices, i.e., choices of lower value options, compared with exploitations. However, when examining patterns within each type of choice, we found that pupil size was affected by task parameters only during exploitative choices. Both choice horizon and value-gap significantly predicted average pupil size: pupils were larger when only one decision remained, reflecting how importance and value of control shape arousal, and when value differences in values were small, reflecting directed exploitation. This dissociation reveals that pupil-indexed arousal selectively tracks the strategic demand for control, beyond differentiating simply between exploitative and exploratory decisions.

These findings prompt a refinement of AGT’s predictions regarding exploration and exploitation. AGT proposes that exploration engages elevated tonic arousal while exploitation engages transient phasic bursts (Aston-Jones and Cohen, 2005; Jepma and Nieuwenhuis, 2011). Our findings partially support this framework: we observed modulation of average (tonic) pupil size in exploratory and exploitative choices, consistent with AGT’s emphasis on sustained arousal states. However, further task-related modulations were observed during exploitation rather than exploration, and specifically during high uncertainty, where exploitative decisions are still uncertain and provide information, similar to directed exploration. This suggests that exploration and exploitation control strategies should be defined according to task demands and not just based on choice value. Moreover, we did not observe discrete phasic peaks time-locked to decisions but instead sustained elevation throughout the decision period. This pattern is consistent with the view that pupil signals reflect context-dependent integration of multiple neuromodulatory and cognitive processes, rather than a one-to-one mapping onto phasic versus tonic LC activity (Joshi and Gold, 2020). Notably this stable temporal profile contrasts with the discrete phasic bursts observed in visual- attentional tasks (Gabay et al., 2011; Preuschoff et al., 2011) and may reflect prolonged evaluative processing under strategic constraints, as was tested in early study in working memory domain (Granholm et al., 1996).

The absence of pupil modulation during exploration warrants consideration alongside prior evidence. Kozunova et al. (2022) found that directed exploration elicited larger pupil dilations compared to exploitation. Critically, their operationalization involved deliberately choosing a known low-payoff option after learning, thereby overriding an established exploitation bias- a process likely engaging conflict-driven control (Shenhav et al., 2013). In contrast, the Horizon Task manipulates environmental factors that promote exploration without imposing strong conflict or immediate costs. Participants can explore with relatively low cost, particularly when horizons are long and future opportunities remain available (Wilson et al., 2014; Zajkowski et al., 2017). This difference in the way exploration is elicited by the task may explain why we observed no pupil modulation during exploration despite systematic exploration strategies, as pupil-linked arousal in such contexts may primarily reflect uncertainty at the level of the environment rather than decision-specific control demands (Fan et al., 2023).

This interpretation aligns with the Expected Value of Control (EVC) framework (Shenhav et al., 2013), which proposes that control is allocated when anticipated benefits outweigh costs. Exploitation under short horizons represents high-importance decisions where errors cannot be corrected, and exploitation with small value differences requires enhanced processing for accurate discrimination. Both conditions increase the expected value of control, warranting greater resource investment. Importantly, pupillary responses have been shown to index the amount of cognitive effort actually invested, rather than objective task difficulty per se, highlighting the distinction between task demands and effortful control allocation (van der Wel and van Steenbergen, 2018). This finding is also consistent with resource-rational principles (Lieder and Griffiths, 2020), which argue that people allocate cognitive resources only when the computational costs are justified. Our current adapted paradigm, characterized by relatively low-cost exploration; initial uncertainty is reduced by forced trials and the stakes of any single choice are low. This creates a condition where exploration is not computationally demanding. Thus, the absence of pupil modulation during exploration may not indicate a failure of strategic processing, but rather an efficient adaptation where costly cognitive control was unnecessary. This account is also in line with recent works that similarly shown that pupillary responses track the efficacy and reward value of control deployment (Frömer et al., 2021) and vary systematically with task demands (Nassar et al., 2012).

Our findings should be interpreted in light of several limitations. The Horizon Task structure provides initial outcome information via forced trials, reducing baseline uncertainty and potentially lowering the strategic demands of directed exploration relative to paradigms requiring information-seeking under complete uncertainty. This limits generalizability to contexts involving substantial opportunity costs or cognitive conflict. Additionally, pupillometry provides an indirect measure of arousal, and individual differences in exploration strategies and cognitive control capacity were not examined. Future work combining pupil measurements with direct neural recordings (e.g., fMRI) across diverse decision context, particularly those where exploration and exploitation vary in strategic importance beyond immediate outcomes- will clarify how pupil-linked arousal indexes context-dependent control allocation. Moreover, from a modeling perspective, although we evaluated more complex random-effects structures, models including random slopes did not converge reliably, likely reflecting overparameterization relative to the experimental design and the limited number of levels per predictor (Bates et al., 2015). We therefore report parsimonious models with random intercepts for participants, highlighting the need for future studies with greater within-subject variability to more fully characterize individual differences in context-dependent control demands.

To conclude, our results provide novel insights into how different styles of control shape pupil-indexed arousal, integrating AGT and EVC theoretical frameworks. We demonstrate that arousal does not uniformly distinguish exploration from exploitation but instead tracks contextual factors determining when control is strategically warranted.

## Data Availability

The datasets presented in this study can be found in online repositories. The names of the repository/repositories and accession number(s) can be found here: https://osf.io/dq3ke/overview?view_only=a3c445dac8bd425fbd114f0047fb1d7a.
