# Supplementary material for: Pupil size response within direct and random exploration and exploitation behaviors selectively reflects value of control
Source: Front Psychol. 2026 Mar 3;17:1752586. doi: 10.3389/fpsyg.2026.1752586 (PMC13040367; doi:10.3389/fpsyg.2026.1752586)
Supplement: Supplementary file 1 [file Table_1.docx]

**Selective Pupil Size Response Within direct and random exploration and exploitation Behaviors**

Gili Barkay^1,2^*, Shai Gabay^1,2^ & Uri Herz^2,3^

**supplementary materials**

1. **Bin Analysis- Trial by trial pupil size**

Linear mixed-effects model analyses for pupil size across the decision window revealed a consistent negative association with value gap, such that larger differences between options were accompanied by smaller pupil diameter. This effect was weak and stable rather than phasic. Bin-wise analyses confirmed this pattern, with negative coefficients for value gap across bins, significant effects of planning horizon at early (Bin –6) and late (Bin –1) pre-choice intervals, and a marginal effect at choice onset (Bin 0). No significant effects were observed for information gap in any bin. Overall, these findings indicate that decision variables modulated pupil-linked arousal in gradual and sustained ways, without evidence for transient peaks.

| **Fixed Coefficients (bin 0= choice)** | | | | | | |
| --- | --- | --- | --- | --- | --- | --- |
| **Model Term** | **Coefficient** | **Std. Error** | **t** | **Sig.** | **95% Confidence Interval** | |
|  |  |  |  |  | **Lower** | **Upper** |
| **Intercept** | 4117.926 | 996.0669 | 4.134 | 0.000 | 2165.174 | 6070.679 |
| **Value Gap** | -1.140 | 0.5761 | -1.978 | 0.048 | -2.269 | -0.010 |
| **Information gap (unequal)** | 0.034 | 10.6900 | 0.003 | 0.997 | -20.923 | 20.992 |
| **equal** | 0^b^ |  |  |  |  |  |
| **Choice Horizon=1** | 18.253 | 10.6867 | 1.708 | 0.08 | -2.698 | 39.204 |
| **Choice Horizon=6** | 0^b^ |  |  |  |  |  |
|  |  |  |  |  |  |  |
|  |  |  |  |  |  |  |
| **Fixed Coefficients (bin- 1)** | | | | | | |
| Model Term | Coefficient | Std. Error | t | Sig. | 95% Confidence Interval | |
|  |  |  |  |  | Lower | Upper |
| **Intercept** | 4116.585 | 995.8497 | 4.134 | 0.000 | 2164.266 | 6068.905 |
| **Value Gap** | -1.014 | 0.5700 | -1.778 | 0.075 | -2.131 | 0.104 |
| **Information gap (unequal)** | -0.927 | 10.5843 | -0.088 | 0.930 | -21.677 | 19.823 |
| **equal** | 0^b^ |  |  |  |  |  |
| **Choice Horizon=1** | 21.797 | 10.5823 | 2.060 | 0.039 | 1.051 | 42.544 |
| **Choice Horizon=6** | 0^b^ |  |  |  |  |  |
| **Fixed Coefficients (bin -2)** | | | | | | |
| **Model Term** | **Coefficient** | **Std. Error** | **t** | **Sig.** | **95%  Confidence Interval** | |
|  |  |  |  |  | **Lower** | **Upper** |
| **Intercept** | 4130.457 | 995.1803 | 4.150 | 0.000 | 2179.340 | 6081.574 |
| **Value Gap** | -1.196 | 0.6239 | -1.916 | 0.055 | -2.419 | 0.028 |
| **Information gap (unequal)** | -4.040 | 11.6401 | -0.347 | 0.729 | -26.861 | 18.781 |
| **equal** | 0^b^ |  |  |  |  |  |
| **Choice Horizon=1** | 14.303 | 11.6376 | 1.229 | 0.219 | -8.514 | 37.119 |
| **Choice Horizon=6** | 0^b^ |  |  |  |  |  |
|  | | | | | | |
|  | | | | | | |
|  |  |  |  |  |  |  |
| **Fixed Coefficients (bin -3)** | | | | | | |
| **Model Term** | **Coefficient** | **Std. Error** | **t** | **Sig.** | **95%**  **Confidence Interval** | |
|  |  |  |  |  | **Lower** | **Upper** |
| Intercept | 4131.510 | 990.5385 | 4.171 | 0.000 | 2189.332 | 6073.688 |
| Value Gap | -1.280 | 0.6910 | -1.852 | 0.064 | -2.635 | 0.075 |
| **Information gap (unequal)** | -1.785 | 12.8462 | -0.139 | 0.889 | -26.973 | 23.402 |
| **equal** | 0^b^ |  |  |  |  |  |
| Choice Horizon=1 | 7.069 | 12.8367 | 0.551 | 0.582 | -18.100 | 32.238 |
| Choice Horizon=6 | 0^b^ |  |  |  |  |  |
| **Fixed Coefficients (bin -4)** | | | | | | |
| **Model Term** | **Coefficient** | **Std. Error** | **t** | **Sig.** | **95% Confidence Interval** | |
|  |  |  |  |  | **Lower** | **Upper** |
| **Intercept** | 4119.764 | 962.3234 | 4.281 | 0.000 | 2232.678 | 6006.849 |
| **Value Gap** | -0.575 | 0.7824 | -0.735 | 0.462 | -2.109 | 0.959 |
| **Information gap (unequal)** | 2.096 | 14.5045 | 0.145 | 0.885 | -26.346 | 30.539 |
| **equal** | 0^b^ |  |  |  |  |  |
| **Choice Horizon=1** | 12.400 | 14.4653 | 0.857 | 0.391 | -15.966 | 40.766 |
| **Choice Horizon=6** | 0^b^ |  |  |  |  |  |
|  |  |  |  |  |  |  |
| **Fixed Coefficients (bin -5)** | | | | | | |
| **Model Term** | **Coefficient** | **Std. Error** | **t** | **Sig.** | **95% Confidence Interval** | |
|  |  |  |  |  | **Lower** | **Upper** |
| **Intercept** | 4139.697 | 958.4222 | 4.319 | 0.000 | 2259.824 | 6019.569 |
| **Value Gap** | -1.349 | 0.9370 | -1.440 | 0.150 | -3.187 | 0.489 |
| **Information gap (unequal)** | -4.132 | 17.3289 | -0.238 | 0.812 | -38.121 | 29.857 |
| **equal** | 0^b^ |  |  |  |  |  |
| **Horizon=1** | 18.842 | 17.3286 | 1.087 | 0.277 | -15.147 | 52.831 |
| **Horizon=6** | 0^b^ |  |  |  |  |  |
| **Fixed Coefficients (bin -6 )** | | | | | | |
| **Model Term** | **Coefficient** | **Std. Error** | **t** | **Sig.** | **95% Confidence Interval** | |
|  |  |  |  |  | **Lower** | **Upper** |
| **Intercept** | **4126.272** | **906.5165** | **4.552** | **0.000** | **2347.591** | **5904.953** |
| **Value Gap** | **-1.349** | **0.9370** | **-1.440** | **0.150** | **-3.187** | **0.489** |
| **Information gap (unequal)** | **-11.700** | **21.2352** | **-0.551** | **0.582** | **-53.366** | **29.965** |
| **equal** | **0^b^** |  |  |  |  |  |
| **Horizon=1** | **51.323** | **21.2758** | **2.412** | **0.016** | **9.578** | **93.069** |
| **Horizon=6** | **0^b^** |  |  |  |  |  |

**B. Distribution of response times (RTs)**

**
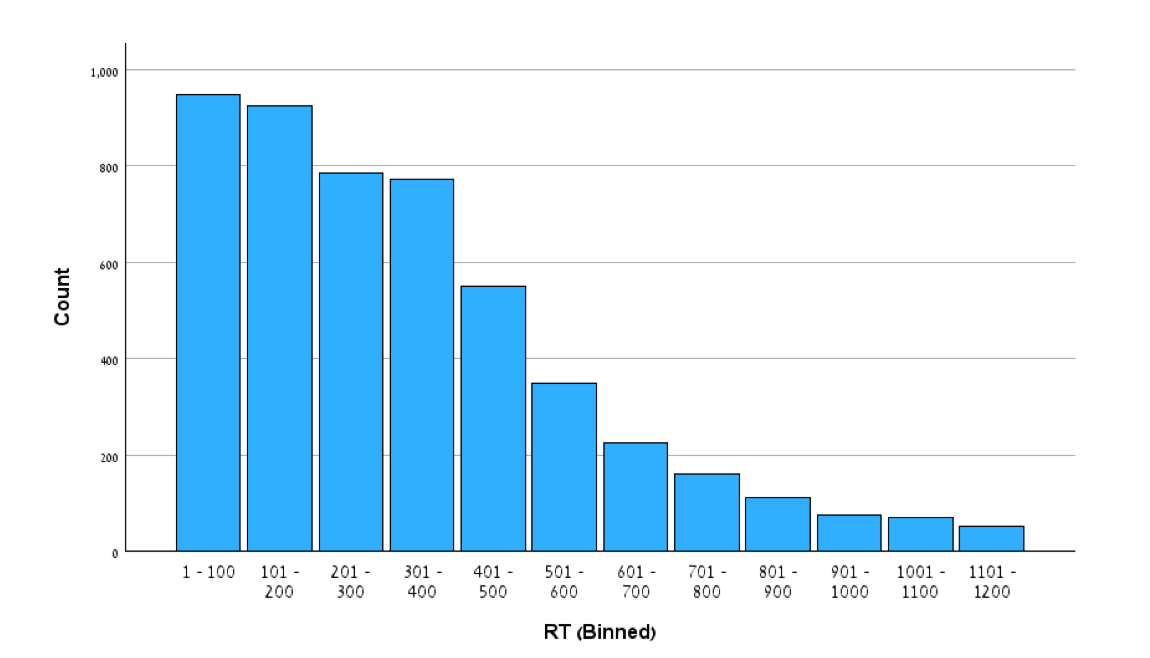
**
